# ErbB2 Receptor Immunoreactivity in Prostate Cancer: Relationship to the Androgen Receptor, Disease Severity at Diagnosis and Disease Outcome

**DOI:** 10.1371/journal.pone.0105063

**Published:** 2014-09-12

**Authors:** Peter Hammarsten, Johanna Winther, Stina H. Rudolfsson, Jenny Häggström, Amar Karalija, Lars Egevad, Torvald Granfors, Christopher J. Fowler

**Affiliations:** 1 Department of Medical Biosciences, Pathology, Umeå University, Umeå, Sweden; 2 Department of Pharmacology and Clinical Neuroscience, Pharmacology, Umeå University, Umeå, Sweden; 3 Department of Surgical and Perioperative Sciences, Urology, Umeå University, Umeå, Sweden; 4 Umeå School of Business and Economics, Department of Statistics, Umeå University, Umeå, Sweden; 5 Department of Integrative Medical Biology, Anatomy, Umeå University, Umeå, Sweden; 6 Department of Pathology and Cytology, Karolinska University Hospital, Stockholm, Sweden; 7 Department of Urology, Central Hospital, Västerås, Sweden; Lund University, Sweden

## Abstract

**Background:**

ErbB2 is a member of the epidermal growth factor family of tyrosine kinases that is centrally involved in the pathogenesis of prostate cancer and several studies have reported that a high expression of this protein has prognostic value. In the present study, we have investigated whether tumour ErbB2 immunoreactivity (ErbB2-IR) has clinically useful prognostic value, i.e. that it provides additional prognostic information to that provided by routine clinical tests (Gleason score, tumour stage).

**Methodology/Principal Findings:**

ErbB2-IR was measured in a well-characterised tissue microarray of tumour and non-malignant samples obtained at diagnosis. Additionally, mRNA levels of ErbB2-IR in the prostate were determined in the rat following manipulation of circulating androgen levels. Tumour ErbB2-IR was significantly associated with the downstream signalling molecule phosphorylated-Akt and with the cell proliferation marker Ki-67. The significant association of tumour ErbB2-IR with the Gleason score at diagnosis was lost when controlled for the association of both parameters with Ki-67. In the rat prostate, mRNA for ErbB2 was inversely associated with circulating androgen levels. There was no association between ErbB2-IR and the androgen receptor (AR)-IR in the tumours, but an interaction between the two parameters was seen with respect to their association with the tumour stage. Tumour ErbB2-IR was confirmed to be a prognostic marker for disease-specific survival, but it did not provide significant additive information to the Gleason score or to Ki-67.

**Conclusions/Significance:**

It is concluded that tumour ErbB2-IR is of limited clinical value as a prognostic marker to aid treatment decisions, but could be of pathophysiological importance in prostate cancer.

## Introduction

ErbB2 (Her-2/neu) is a member of the epidermal growth factor family of tyrosine kinases involved in a variety of cellular responses including apoptosis, migration, growth, adhesion and differentiation [1]. ErbB2 forms heterodimers with other members of the ErbB family and initiates a cascade of signalling pathways, including activation of the survival factor Akt [1], [2]. ErbB2 is also found in tumour cells, and trastuzumab, an ErbB2 monoclonal antibody, is used for the adjuvant treatment of ErbB2-positive breast cancer. Other ErbB2 monoclonal antibodies are also under clinical development [3].

The importance of ErbB2 in the pathogenesis of prostate cancer (Pca) is less clear. Pca growth is regulated by androgen receptors (AR), and a key treatment of androgen-dependent Pca is a reduction of androgen levels either by surgical or medical castration. However, the effect of the treatment is temporary, and the cancers become androgen-resistant. In 1999, Craft et al. [4] reported that introduction of ErbB2 into LNCaP androgen-dependent Pca cells rendered them partially resistant to the growth-inhibition produced by androgen deprivation. Further, in a xenograft model using castrated mice, the ErbB2-expressing cells produced a more rapid tumour formation than the untransfected LNCaP cells [4]. The predictive validity of xenograft models is controversial [5], but an increased rate of metastasis has been reported in an orthotopic model of Pca following injection into the ventral prostate of nude mice of NbE-1.4 rat prostate cells overexpressing the rat homologue of ErbB2 [6]. Conversely, treatment of female BALB/c nude mice with an ErbB2 antibody reduced tumour growth following injection of 22Rv1 Pca cells [7]. Removal of androgens from the cell culture medium increases ErbB2 expression [8], and a slight increase in ErbB2 expression seven days after castration is seen in a xenograft model of tumour growth with CWR22 cells [9].

While the studies in cell lines and animal models point to a key role of ErbB2 in the pathogenesis of Pca, studies in humans are less clear. Different studies have reported very large variations in the levels of ErbB2 expression in prostate tumour tissue (see [10] and references therein). However, a meta-analysis of the literature between 1996 and 2009 found an increased relative risk for death due to Pca or biochemical (prostate-specific antigen, PSA) recurrence of the disease in patients with moderate to high levels of tumour ErbB2 expression [11]. These authors concluded that “further clinical trials should test the hypothesis that HER-2/neu is a marker of a clinically worse outcome in patients with prostate cancer and a potential target for therapy”. One such subsequent investigation in a large (>1700) number of patients confirmed that the prognostic properties of ErbB2 overexpression with PSA recurrence as end-point, although it did not provide additive prognostic information to that given by the Gleason score, the tumour stage and the preoperative PSA level [10]. Although the patients were not treated during the follow-up period, the samples were obtained at radical prostatectomy rather than at diagnosis. It is thus important to determine whether ErbB2 has a better prognostic value (i.e. provides additive information to that given using the Gleason score and other prognostic markers) in a cohort of patients where the tissue samples were obtained at diagnosis and the natural progression of the disease can be studied. This has been investigated in the present study.

## Materials and Methods

### Ethics statement

The human material was collected according to Swedish regulations at a time when informed consent was not required. The research ethical committee at Umeå university hospital (Regional Ethical Review Board in Umeå) approved of the study (no. 02-283) and waived the need for consent.

Ethical permission (no. A110-12) for the animal experiments was obtained from the local animal ethical committee (Umeå Ethics Committee for Animal Studies, Sweden).

### Patient samples

The cohort used is from a consecutive series of 412 men who were diagnosed with prostate cancer following transurethral resection for lower urinary tract symtoms [12]. The cases were collected between 1975 and 1991 at the Central Hospital, Västerås, Sweden, a time period when PSA testing was not routine. Since the patients were diagnosed post-operation, they had not received hormonal treatment or radiotherapy before transurethral resection. The samples were then formalin-fixed and paraffin-embedded. Using cores (diameter 0.6 mm) the tissue microarrays were made using a Beecher instrument (Sun Prairie, WI, USA). For each patient, up to eight cores (usually 5) of tumour tissue and up to four samples of non-malignant tissue from each patient were placed [12].

### ErbB2 immunoreactivity (ErbB2-IR) in Pca microarrays

The 4 µm paraffin-embedded tissue micro array sections were placed on slides, baked at 60°C, 1 h then deparaffinised and rehydrated. Peroxide block with 3% H_2_O_2_ in methanol for 20 min was undertaken. Antigen retrieval was performed with sections placed in a citrate buffer pH 6.0 and steamed in a microwave oven for 2 min. The solution was allowed to cool for 5 min, washed in distilled water and then TBS buffer. The protein-block (DAKO, Stockholm, Sweden) was performed for 15 min after which the sections were exposed to the primary monoclonal antibody (mouse c-erbB-2 Prediluted Cocktail Antibody (PM070AA), Biocare, CA, USA) for 2 h in RT. Then the secondary antibody (horse anti mouse (BA-2000), Vector Laboratories, Burlingame, CA, USA) was incubated at 30 min at RT followed by avidin-biotin complex tagging for 30 min at RT using the Elite ABC Kit (Vector Laboratories) and lastly DAB (DAKO) enhancement for 40 s at RT.

Tumour (n = 1822) and non-malignant (n = 1170) cores were scored for ErbB2 in prostate epithelial cells and tumour cells from digitally scanned images by one evaluator (JW) who did not at the time of the evaluation have access to the clinical data. The tumour samples were scored on basis of immunoreactive intensity (ranging from 0 =  no staining to 4 = maximal staining) and distribution (0, 25, 50, 75, 100%). Note that the distribution scores did not include stroma, since this did not express ErbB2-IR. Thus, a sample with only a small proportion of epithelial cells but with a large amount of stroma can still score a distribution of 100% and an intensity of 4. Then, for each core a composite value was determined (between 1–4). For example, a core with where 25% of the scorable sample had intensity 2 and 75% had intensity 3 would receive a composite score of (0.25×2+0.75×3) = 2.75. Median values were then determined for each case. To determine consistency, 301 tumour cores were scored and then rescored. Interclass correlation coefficient for consistency (mixed model) gave Cronbach's alpha of 0.90, 254 of 301 scores were within 0.5 score units of each other. The non-malignant cores were additionally scored for basal and luminal cells separately, when possible. Some cores (n = 482) had clearly identifiable basal and luminal cells and these have been returned as basal and luminal in the database. A diffuse basal layer (n = 436) was seen in some other cores and in the remaining cores (n = 252) basal cells could not be identified. These cores were not used here, so it should be remembered as a caveat that the scores for basal and luminal non-malignant ErbB2-IR represent a limited dataset. Ki67 index, pAkt-IR, androgen receptor (AR)-IR and the clinical characteristics for each patient were available in the database and have been reported in whole or in part previously [12]–[15].

### Animals and treatments

Forty-two adult male Sprague-Dawley rats (284–430 g) were anesthetized with pentobarbitalnatrium (50 mg/kg) and castrated via the scrotum. Six intact animals served as controls. After 7 days, twenty-one of the animals received a subcutaneous injection of long acting testosterone (5 mg/kg, Sustanon, Organon, Oss, The Netherlands) given as a single dose every second day as earlier described [16]. Prostate tissue were removed at different times after castration (1, 3, 7 days) and at different times after castration + testosterone treatment (1 day, 2 days, 3 days) and frozen in liquid nitrogen. There were seven groups including the control group and each group consisted of six animals.

The experimental design of this study proceeded according to the guidelines for care and management of laboratory animals and was approved by the local animal ethical committee (Umeå Ethics Committee for Animal Studies, Sweden). Animals were housed under a controlled temperature in an artificially illuminated room (12-h light/12-h dark). Food and tap water were freely available.

### RNA preparation and Quantitative Reverse transcription PCR

Total RNA from tissues was isolated according to the TRIzol method (Invitrogen, Stockholm, Sweden). RNA concentrations were quantified spectrophotometrically at 260 nm (DU 640 Spectrophotometer, Beckman Coulter, Bromma, Sweden) and RNA integrity was verified by 28 S and 18 S rRNA integrity after agarose gel electrophoresis (data not shown).

Five hundred ng total RNA was reversed transcribed using random hexamers (Applied Biosystems, Sundbyberg, Sweden) and Superscript II reverse transcriptase (Invitrogen, Stockholm, Sweden) in a 10 µl reaction according to the manufacturer's instructions. All cDNA reactions were run in duplicates.

Quantification of ErbB2 mRNA was performed by real-time quantitative PCR using the ABI PRISM 7900HT Instrument (Applied Biosystems, Inc., Foster City, CA). Reactions were run in a 20 µl volume using TaqMan gene expression assays Rn00566561_m1 (Applied Biosystems, Life Technologies Europe BV, Stockholm, Sweden) with the TaqMan gene expression mastermix according to manufacturer's protocol. Each sample was analyzed for Rpl13a (Rn00821946_gl, Applied Biosystems) and β-actin (Rn00667869_ml, Applied Biosystems) expression to use as a normalizing reference controls. To confirm amplification specificity the PCR products were subjected to a melting curve analysis. The quantification data were analysed with SDS 2.4 Software (Applied Biosystems) and the relative values of ErbB2 mRNA in the samples are calculated from a standard curve obtained by amplification of five-fold serial dilution of reversed transcribed total RNA from a reference sample. An issue in this type of study is the appropriate housekeeping to use, since their levels can vary in cancer research studies [17], [18]. We have elected to present the data normalized to two genes: ß-actin and rpl13a, in order to give an assessment of the robustness of the data.

### Statistics

The intraclass correlation coefficient, binary logistic and Cox proportional-hazards regression analyses were conducted using SPSS software (IBM). Ordinal regression analyses utilised software developed within the R project for statistical computing (version 2.15.2) [19]. All other statistical calculations used the statistical package built into the GraphPad Prism 6 computer programme for the Macintosh (GraphPad Software Inc., San Diego, CA, USA). For cases followed by expectancy, Receiver Operating Characteristic (ROC) curves with a 15-year follow-up limit were used to define cut-offs for ErbB2-IR. For these curves and for the Kaplan-Meier survival curves, an event was defined as death attributable to prostate cancer and registered into the database as “event = 1”. Patients with death from other causes and cases where the patients were alive at the time of follow-up were censored and entered into the database as event  = 0.

## Results

### ErbB2-IR in Pca tissue microarrays: comparison of tumour and non-malignant scores

**Figure 1:**
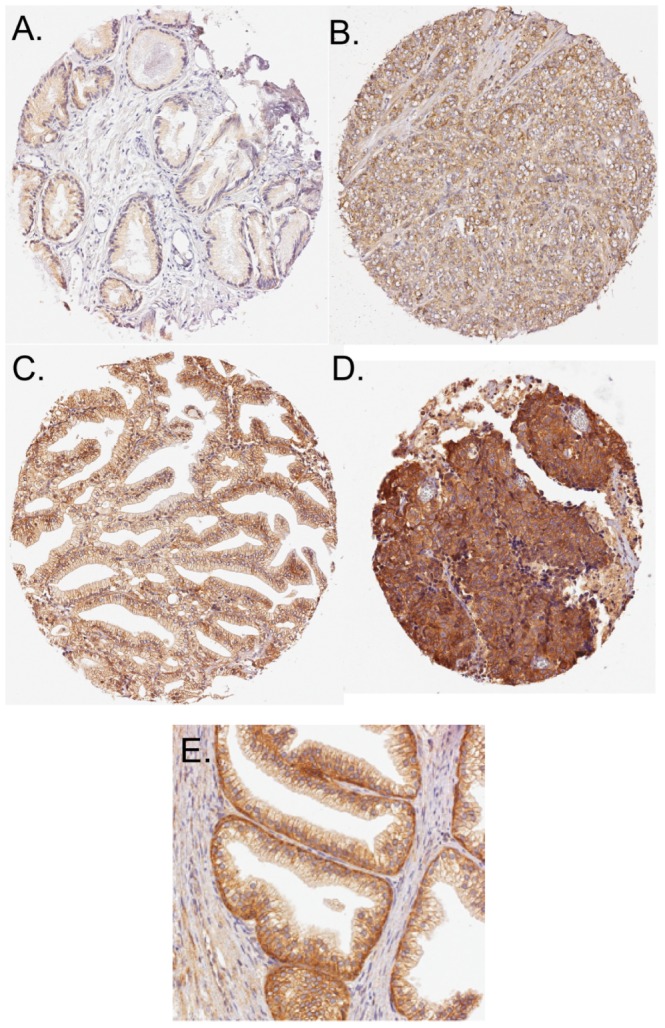
Examples of tissue microarray tumour cores with different ErbB2 immunoreactivities (A–D). The assigned scores were: Score 1 for Panel A; Score 2 for Panel B; Score 2.25 (75% intensity score 2, 25% intensity score 3) for Panel C; Score 4 for Panel D. The Gleason scores of the cases from which the cores were derived were 5 for Panels A and C, and 10 for Panels B and D. Panel E shows example of ErbB2 immunoreactivity in a non-malignant core, where basal and luminal staining could easily be identified.

**Figure 2:**
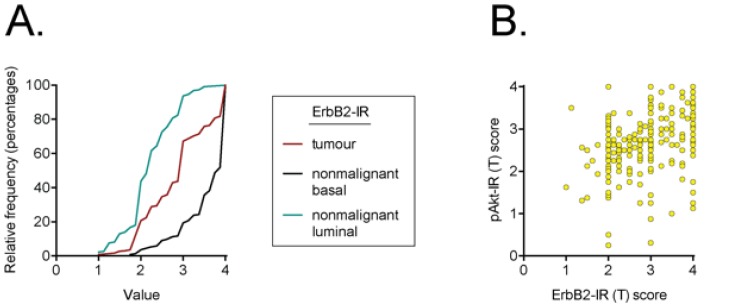
ErbB2 immunoreactivity in Pca tissue microarrays. In Panel A the cumulative frequencies of ErbB2-IR scores are shown for the tumour samples (n = 357), and for the basal and luminal nonmalignant samples (n = 222 in both cases). The median values (with interquartile range in brackets) were: tumour, 3.0 (2.25–3.5); luminal nonmalignant, 2.25 (2.0–2.625); basal non-malignant 3.875 (3.5–4.0). The median values were significantly different from each other (p<0.001, Dunn's multiple comparison test following significant Kruskal-Wallis test). Panel B shows a scatterplot of the individual scores for tumour ErbB2-IR and pAkt-IR (taken from [14]).

A total of 357 (tumour) and 222 (non-malignant basal and luminal) cases were scored for ErbB2-IR. Examples of different ErbB2-IR intensities from four tumour cores are shown in Fig. 1A–D. An example of non-malignant tissue showing a clear difference between basal and luminal ErbB2-IR is shown in Fig. 1E. For both the tumour and non-malignant samples, the epithelial cells showed ErbB2-IR whereas stroma did not. The cumulative scores for the tumour epithelial and the non-malignant basal and luminal cells are shown in Fig. 2A. For matched samples, the scores were significantly different from each other (P<0.0001 for pairwise tests, Wilcoxon matched-pairs signed rank test). At the level of individual cores, the number of cases with basal scores ≥−1, −0.99 to −0.01, 0, 0.01 to 0.99, 1 to 1.99 and ≥2 units higher than the matched luminal scores were 1, 1, 5, 86, 286 and 103, respectively.

### Association of ErbB2-IR with disease severity at diagnosis

The association of ErbB2-IR with clinical (age, Gleason score, % of core that was tumour associated) and biochemical (Ki67 index, stroma AR-IR and pAkt-IR) measures within the same region are shown in Table 1. First-order Spearman's rho values in Table 1 were calculated as described in [20]. In the tumour samples, significant associations with the Gleason score, % of core that was tumour associated, Ki67 index and pAkt-IR were found. A scatterplot showing the association between tumour ErbB2-IR and pAkt-IR scores is shown in Fig. 2B. Consistent with the significant Spearman's rho value, the distribution of cases with Gleason scores 4–5, 6, 7 and 8–10, respectively, was different for those with a tumour ErbB2-IR score ≤2.75 (40 [26%], 46 [30%], 26 [17%] and 41 [27%]) than those with a score >2.75 (21 [10%], 57 [28%], 40 [20%] and 86 [42%]) (P<0.0005, Chi squared test; values given are the number [% for the ErbB2-IR group] with the Gleason score range at diagnosis. The corresponding values for the tumour grades 1a-1b, 2, 3 and 4, respectively, were: tumour ErbB2-IR score ≤2.75, 92 [61%], 30 [20%], 25 [17%] and 3 [2%]); tumour ErbB2-IR score >2.75, 83 [41%], 61 [30%], 49 [24%] and 8 [4%] (P<0.005, χ^2^ test).

**Table 1:** Correlations of Pca tissue microarray ErbB2-IR scores with clinical and biochemical measures.

	Zero-order correlations			First-order correlations (controlling for Ki67)		
Parameter	r	n	P-value	r	n	P-value
*Tumour ErbB2-IR*						
Age	−0.051	357	0.34			
GS	0.191	357	0.0003	0.059	350	0.27
%ca	0.111	357	0.036	0.000	350	1
Ki67 index (T)	0.287	350	<0.0001			
Epithelial AR-IR (T)	−0.040	310	0.49			
pAkt-IR (T)	0.363	271	<0.0001	0.256	267	0.0001
*Basal N ErbB2-IR*						
Age	−0.005	222	0.94			
GS	−0.112	222	0.096			
%ca	−0.121	222	0.072			
Ki67 index (N)	0.119	219	0.080			
Epithelial AR-IR (N)	0.137	205	0.050	0.122	205	0.081
pAkt-IR (N)	0.166	173	0.029	0.168	171	0.029
*Luminal N ErbB2-IR*						
Age	0.040	222	0.56			
GS	−0.028	222	0.68			
%ca	0.031	222	0.64			
Ki67 index (N)	0.093	219	0.17			
Epithelial AR-IR (N)	0.112	205	0.11			
pAkt-IR (N)	0.142	173	0.063	0.149	171	0.052

Abbreviations: r, Spearman's rho; GS, Gleason Score; %ca, % of core that was tumour associated; T, tumour; N, non-malignant. First-order Spearman's rho values were calculated as described in [20].

In theory, a significant association of two parameters could be due to the fact that both parameters are associated with a third parameter. Thus, for example, if cases with a high rate of cell proliferation, as seen with the marker Ki67 index, more often have high Gleason scores and higher ErbB2-IR scores than cases with low rates of cell proliferation, then a correlation between ErbB2-IR and the Gleason scores would be induced. Consistent with this situation, the first-order correlations between ErbB2-IR and either the Gleason score or % of core that was tumour associated controlling for the tumour Ki67 index were not significant. However, the association between tumour ErbB2-IR and pAkt-IR remained significant (Table 1). Thus, in the tumour tissue, ErbB2-IR scores are associated with the downstream signalling molecule pAkt and with the cell proliferation marker Ki67, but not robustly with the clinical measures of disease severity. No significant correlation between ErbB2-IR and either the Gleason score, the % of core that was tumour associated or the non-malignant Ki67 index was seen for either basal or luminal ErbB2-IR. Significant associations between basal ErbB2-IR and both non-malignant pAkt-IR and stroma AR were seen.

### Relationship between tumour ErbB2 and androgen signalling

Ricciardelli et al. [21] reported that high levels of both tumour ErbB2-IR and AR-IR were more common in Pca patients with stage 3 tumours (n = 31) than in stage 2 tumours (n = 22, samples obtained at radical prostatectomy). Here we investigated the relationship between these two variables in our tissue microarray. Consistent with the study of [21], there was no correlation between the two variables *per se* in the tumour tissue (Table 1). However, in our material, cases with levels of both tumour ErbB2-IR and AR-IR above the median were less common in patients with stage 3 tumours (n = 58) than for stage 2 tumours (n = 82) (Table 2). As to be expected from the correlation analyses shown in Table 1, a confounding issue is that the Ki-67 index varies between the groups (Table 2).

**Table 2:** Tumour stages for patients with different tumour epithelial ERBb2-AR and AR-IR scores.

	AR-IR <50%		AR-IR ≥50%		
	ErbB2-IR <3	ErbB2-IR ≥3	ErbB2-IR <3	ErbB2-IR ≥3	P-value
*Tumour stage*					
1a-1b	30 (45%)	32 (37%)	56 (74%)	39 (49%)	<0.0001
2	14 (21%)	24 (28%)	15 (20%)	29 (37%)	
3	21 (32%)	26 (30%)	3 (8%)	8 (10%)	
4	1 (2%)	4 (5%)	2 (3%)	3 (4%)	
*Ki67 index*	2.6 (n = 66)	3.5 (n = 86)	1.8 (n = 75)	2.9 (n = 77)	<0.0001
	[1.1–4.3]	[1.5–8.9]	[0.6–3.2]	[1.5–5.9]	

Values shown for the tumour stage are the number of cases, with the percentage of the total for the given ErbB2-IR/AR-IR group. Values shown for the Ki67 index are the median scores, with sample sizes and interquartile ranges in brackets. The divisions represent a median split of each parameter. AR-IR is given as the % of cells with nuclei staining positive for this receptor (see [15]). The P value was determined by a χ^2^ analysis of the data (for tumour stage) or by Kruskall-Wallis one way analysis of variance by ranks (for Ki-67 index). P<0.0001 indicates a significant variation between the four groups defined on the basis of their AR-IR and ErbB2-IR scores.

In order to account for this, we undertook ordinal regression analyses with tumour ErbB2-IR, AR-IR and Ki67-index as the independent variables and the tumour stage as the dependent ordinal variable. As a caveat, it should be pointed out the Ki67-index correlates both with ErbB2-IR (Table 1) and AR-IR (Spearman's rho -0.17, n = 340, P<0.005) and so the three variables are not truly independent. However, the correlations, although significant, are relatively low, so the risk of multicollinearity is small. Further, the word “ordinal” in ordinal regression analysis refers to the dependent variable, and the values of the independent variables have been defined as continuous, rather than ordinal, which is in fact the case for the scores (hence, for example, the use of Spearman's rho rather than Pearson's r in Table 1). However, this is entirely reasonable in ordinal regression models when the variable in question has a large number of categories (there are 22 different tumour ErbB2 scores ranging from 1 to 4 in the database). In the main effects model, the values returned from the ordinal regression analysis for a variable represent its total effect when the other variables are included. In this case, both the Ki67 index (positive) and the AR-IR (negative) were significant, whereas ErbB2 was not (Table 3). In the interaction term model, the value for the individual variable is for a constant value of the other variables. In this case, the results for the three variables were the same in terms of significance, however a significant interaction ErbB2-IR x AR-IR (positive) was returned. In other words, when variables are held constant, there is an influence of the combination of ErbB2-IR x AR-IR, so that the higher the scores, the higher the tumour stage number. The logit and odds ratio values for the four levels of response (level 1 being the reference level) at different ErbB2-IR and AR-IR scores are shown in Table S1 to illustrate this point for those readers well acquainted with this statistical method.

**Table 3:** Ordinal regression analyses with tumour ErbB2-IR, AR-IR and Ki67-index as the independent variables and the tumour stage as the dependent variable.

	Interaction model			Main effects model		
	Estimate	Z-value	P-value	Estimate	Z-value	P-value
ErbB2-IR	−0.65±0.39	−1.66	0.097	0.12±0.16	0.75	0.45
AR-IR	−0.074±0.023	−3.18	0.0015	−0.027±0.0056	−4.75	<0.0001
Ki67 index	8.27±2.14	3.86	0.0001	7.7±2.1	3.61	0.0003
ErbB2-IR x AR-IR	0.016±0.0076	2.11	0.035			

Two models are shown, one with an interaction between ErbB2-IR and AR-IR (“Interaction model”) and one without this interaction (“Main effects model”). The values returned for a variable in the main effects model represent its total effect when the other variable is included. In the interaction term model, the value for the individual variable is for a constant value of the other variable. The coefficients (± standard error) were obtained from ordinal regressions (cumulative logit model) using the R statistical package (function vglm in the VGAM bundle). The tumour stages were divided into 1a+1b, 2, 3 and 4. In both cases, the assumption of proportional odds was upheld. The models return four intercept coefficients for each level of the response with the reference level being the lowest level. These were as follows: main effects model, 0.61, −0.82, −3.14; model with interaction, 2.84, 1.40, −0.92.

**Figure 3:**
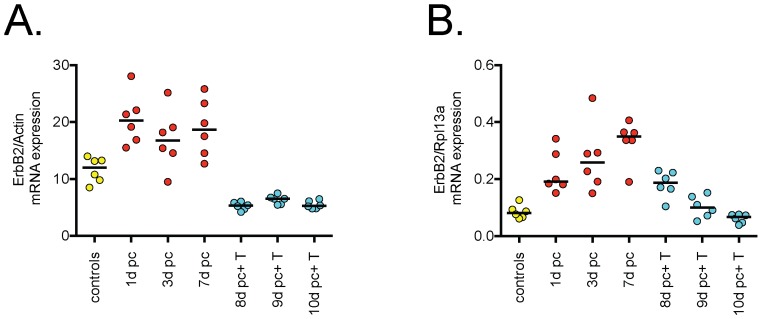
Relative expression of ErbB2 in the rat prostate following castration and androgen reinstatement. Data are normalized to A. ß-actin; B, Rpl13a. Abbreviations: d pc, number of days post-castration; T, testosterone. Shown are the individual values (n = 6 per group); the solid bars indicate medians. In both cases, Kruskal-Wallis tests indicated a significant variation between the groups (P<0.0001).

The data of Ricciardelli et al. [21] and the present study are consistent with, but not proof of, an interaction between androgen signalling and ErbB2 expression. Such proof requires the use of experimental models. Androgen removal affects ErbB2 expression in tumour cell lines [8] and in xenograft models [9]. It is not known, however, whether this regulation is restricted to tumour cells or is also seen in intact prostate tissue following large-scale variations in circulating testosterone levels. In order to determine whether similar regulatory effects were seen in the normal prostate, ErbB2 expression at the mRNA level in this tissue was measured by qPCR following castration in rats and thereafter reinstatement of circulating androgen levels by testosterone injection. The observed results (Fig. 3) are consistent with the tumour studies cited above.

### Prognostic value of tumour ErbB2-IR

A total of 255 cases scored for tumour ErbB2-IR were followed by expectancy after diagnosis, this being the standard treatment at the time. In these cases, the natural progression of the disease can be followed, and the influence of a biomarker upon outcome can be investigated. In general, a cut-off value of the biomarker is chosen, and the outcome is compared between cases below and above the cut-off is determined in survival analyses, such as Cox proportional hazards regression analysis and Kaplan-Meier plots. Cut-off values can be arbitrary, such as a simple median split of the data, or defined upon the basis of a statistical analysis. One such approach is the use of Receiver Operating Characteristic (ROC) analyses, which plot for all possible cut-off values the true positive rate (sensitivity) vs. the false positive rate (1-specificity). For a ROC curve with an area under the curve [AUC] significantly greater than 0.5 (no prognostic value), the optimal cut-off value (the Youden index, representing the cut-off that gives the highest value for sensitivity + specificity – 1) can be calculated (see [22] for description). A ROC curve with a 15-year limit indicated a significant prognostic value of tumour ErbB2-IR (Area under the curve [AUC] 0.60, P<0.05), and was at the division ≤2.75 and >2.75. By comparison, the ROC area under the curve for the tumour Ki67 index was 0.75 (P<0.0001) and so a value of 0.60, albeit significant, is not that impressive. The non-malignant basal and luminal ErbB2-IR scores lacked prognostic value (AUC≤0.55, P≥0.4).

In theory, it is possible that although the AUC for tumour ErbB2-IR is modest, it could contribute to a combination of markers with useful prognostic value. In order to investigate this possibility, two approaches have been taken, using the tumour ErbB2-IR together with three other tumour markers (Ki67 index, epithelial AR-IR and pAkt). The data have been normalised so that the maximum score in each case is 100%, and in the case of AR, the scores have been expressed as 100-normalised score, since a low, rather than a high, AR-IR is associated with a poor prognosis [15]. Different combinations were tested, but none gave an improvement to that seen with Ki67 alone (Table S2). The ROC curves gave equal weighting to the different biomarkers, and so in theory an optimum weighting might have been missed. In consequence, a binary logistic regression analysis with the clinical outcome as dependent variable was investigated using the same parameters. The best combination of parameters in terms of correctly predicting death due to Pca was ∼∼23% and the inclusion of ErbB2 did not improve this figure (Table S3). By comparison, the Gleason score had a prediction rate of 55.6% (Nagelkerke R^2^ value 0.419) on its own and 45.6% (Nagelkerke R^2^ value 0.401) in combination with AR-IR + Ki67-IR (data not shown).

**Figure 4:**
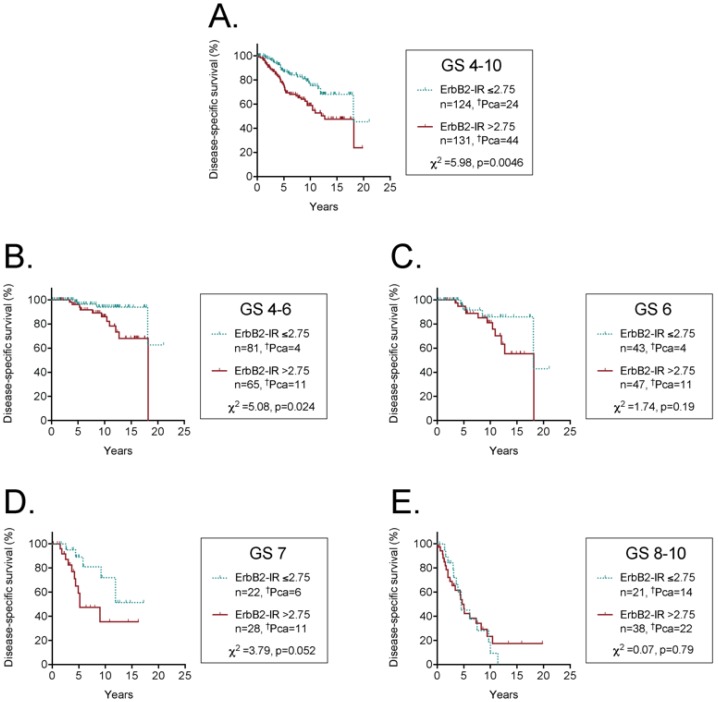
Kaplan-Meier plots of disease-specific survival of patients followed with active expectancy with tumour ErbB2-IR scores either below or above the optimal (Youden) cut-off value. The number of cases where death was due to prostate cancer are indicated as ^†^Pca in the figure. The hatches on the lines show censored data (i.e. cases other than death due to prostate cancer). The range of Gleason scores (GS) for the patients are shown in each panel.

A univariate Cox proportional hazards regression analysis with disease-specific survival as end-point indicated that the relative risk for cases with a tumour ErbB2-IR score above the optimal cut-off, as defined by the Youden index, was approximately double that for those below the cut-off value (the Exp(B) value shown in Table 4). The univariate analysis does not take account of any potential confounding variables. From the previous results presented in this paper, Ki67 is an obvious confounder, and a Bivariate Cox proportional hazards regression analyses indicated that the tumour ErbB2-IR did not provide additional prognostic information to that provided by either the tumour Ki67 index (Table 3). In terms of clinical usefulness, a marker would be of interest if it provided information over and above that provided by the Gleason score. However, a Bivariate Cox proportional hazards regression analyses controlling for Gleason score (three groups: 4–6, 7 and 8–10) indicated that the prognostic value of tumour ErbB2-IR no longer remained significant (Table 4). Consistent with this, Kaplan-Meier plots for all cases followed by expectancy showed a significant prognostic effect of the tumour ErbB2-IR (Fig 4A), and for cases with Gleason scores 4–6 (Fig. 4B). However, a significant prognostic effect was not seen for cases with Gleason scores 6 alone (Fig 4C), Gleason scores 7 alone (Fig 4D, albeit bordering on significance) or for cases with Gleason scores 8–10 (Fig. 4E).

**Table 4:** Cox proportional hazards regression analyses for tumour ErbB2-IR in cases followed by expectancy after diagnosis.

	No. of cases below/above cut-off	Exp(B)	95% CI		P
			Lower	Upper	
*Univariate*					
ErbB2-IR (T)	124/131	2.02	1.23	3.33	0.0056
Gleason score 7	188/53	6.02	3.08	11.74	<0.0001
Gleason score 8–10	188/63	15.86	8.80	28.60	<0.0001
Ki67 index (T)	157/126	4.48	2.62	7.67	<0.0001
pAkt-IR (T)	101/103	3.17	1.77	5.70	0.00011
*Bivariate*					
ErbB2-IR (T)	124/131	1.51	0.91	2.52	0.11
Gleason score 7	146/50	4.86	2.42	9.77	<0.0001
Gleason score 8–10	146/59	11.16	6.01	20.71	<0.0001
ErbB2-IR (T)	122/127	1.49	0.89	2.49	0.13
Ki67 index (T)	135/114	3.53	2.02	6.14	<0.0001
ErbB2-IR (T)	100/94	1.48	0.83	2.65	0.19
pAkt-IR (T)	93/101	2.52	1.36	4.69	0.0034
*Multiivariate*					
ErbB2-IR (T)	99/92	1.09	0.60	1.98	0.79
Gleason score 7	104/39	3.86	1.58	9.43	<0.0001
Gleason score 8–10	104/48	9.68	4.23	22.14	<0.0001
Ki67 index (T)	99/92	1.78	0.86	3.67	0.12
pAkt-IR (T)	92/99	1.60	0.85	3.02	0.15

The Exp(B) value indicates the increased risk as the score goes from below the cut-off to above the cut-off score. Cut-off values for the parameters were as follows: tumour (T) ErbB2-IR ≤2.75 and >2.75; tumour Ki67 index <2.45% and >2.45%; tumour pAkt-IR, <2.75 and ≥2.75. The Exp(B) for Gleason score 7 is relative to 4–6, while the Exp(B) for Gleason scores 8–10 are relative to 4–6. P values indicate whether or not the Exp(B) values are significantly different from unity, i.e. no change in relative risk compared to the values below the cut-off.

## Discussion

In the present study, we have investigated the association of ErbB2-IR with disease severity at diagnosis and with disease outcome in a well-characterised tissue microarray [12]. There is a large body of work concerning the prognostic value of ErbB2 in Pca (review, see [11]), but the present study provides novel information in two important respects: first, the data are obtained in cases who had not received any cancer treatment before the samples were taken, since they were diagnosed only after transurethral resection for lower urinary tract symptoms. Additionally, since the standard treatment at the time was expectancy, the long follow up used allows examination of the natural course of the disease. This is extremely valuable for evaluating biomarkers. Secondly, the availability of data upon AR in the same samples has allowed an examination of the inter-relationships between ErbB2 and AR expression in the tumors. In general, our findings are in good agreement with the literature. With respect to differences in the tissue expression of ErbB2, Lyne et al. [23] reported that the percentage of specimens with moderate to strong ErbB2 immunoreactivity was 100 for benign prostate basal cells, 11 for benign prostate luminal cells, 0 for benign prostate stroma, 87 for prostatic intraepithelial neoplasia and 82 for prostate cancer. In the present study, we did not observe ErbB2-IR in the stroma, and the number (with % in parentheses) of cases with ErbB2-IR scores ≥3 were 196/222 (88%), 39/222 (18%) and 201/357 (56%) for non-malignant basal, luminal and tumour samples, respectively.

In the tumour samples, ErbB2-IR was significantly associated with both the cell proliferation marker Ki67 and the immunoreactivity of pAkt, the phosphorylated form of the survival factor Akt. The association with the Ki67 is consistent with a large study using samples obtained at radical prostectomy, where the average Ki67 labelling index was higher for the 388 ErbB2-positive tumour samples than for the 1940 ErbB2-negative samples (6.1 vs. 4.3, respectively, [10]). Other smaller studies, however, have not reported an association with Ki67 [24], [25]. The Ki67 index is strongly associated with the Gleason score, but the association between the Ki67 labelling index and ErbB2-IR remained when Gleason score subsets were analysed [10]. The association between ErbB2-IR and pAkt-IR, which is to be expected given that Akt is a downstream signalling molecule for ErbB2 [26], was also found in a series of 209 Pca tissue samples obtained at transurethral resection of the prostate [27]. Recently, Le Page et al. [28] found a strong association (r = 0.56, n = 64) between ErbB2-IR and Akt2-IR in a tissue array of samples obtained at radical prostatectomy. The authors also demonstrated that transfection of androgen-independent PC-3 cells with ErbB2 increased Akt2 expression, whereas knockdown of ErbB2 in LNCaP decreased its expression [28].

There was a weak association of tumour ErbB2-IR with the Gleason score at diagnosis, consistent with the literature. Thus, in their meta-analysis of data taken from 38 papers involving 5,976 patients, Neto et al. [11] found that the percent of cases with a Gleason score >7 was higher in the group of cases with a standardised immunostaining of moderate (+2) or high (+3) compared with those with immunostaining of 0 or weak (+1). Although significant, the size of the difference was relatively small (54% vs. 47.6%, [11]), and our analysis indicates that the significant association is lost in first-order correlations controlling for the highly significant association between ErbB2 and the cell proliferation marker Ki67. This would suggest that the association of ErbB2 and the Gleason score is an indirect result of the fact that both these parameters are well correlated with the Ki67 index.

As part of the study, we investigated the association between ErbB2 and androgen signalling. The finding that ErbB2 expression in the normal rat prostate is inversely related to circulating androgen levels is consistent with studies in cultured Pca cells and Pca tumour xenograft models [8], [9]. Given that an important role of ErbB2 signalling is to increase AR transactivation [4], [29], this inverse relationship makes good biological sense, since it means that it minimises fluctuations of AR responses in the face of fluctuations in circulating androgen levels, such as those seen at different times of the day [30]. However, in terms of treatment of Pca, the inverse relationship may be problematical. If it is assumed for the sake of argument that a similar inverse relationship holds in man, then a case could be made for treatment with an ErbB2 monoclonal antibody such as trastuzumab following castration. However, Phase II clinical studies with trastuzumab in castration-resistant Pca patients have been disappointing [31].

With respect to the androgen receptor itself, we did not see an association between AR-IR and ErbB2-IR, in agreement with the study of Ricciardelli et al. [21]. Those authors also reported an interaction between AR-IR and ErbB2-IR with respect to the tumour stage, but not the Gleason score. Thus, 9% of cases with tumour stage 3 had low expression levels of both AR-IR and ErbB2-IR, whilst 73% of the cases had high expression levels of both parameters. For tumour stage 2, the corresponding values were 16 and 35%, respectively. In our dataset, the corresponding values were for tumour stage 2, 17% (14/82 cases) and 35% (29/82); tumour stage 3, 36% (21/58) and 14% (8/58), respectively (data from Table 2). Thus, while the data from the two studies are in good agreement for tumor stage 2, they diverge considerably for tumour stage 3. An additional complicating factor is the association between ErbB2-IR and the Ki67 index, given that the rate of cell proliferation is significantly higher for cases with tumour stage 3–4 than tumour stage 2 in the tissue microarray [13]. However, when ordinal regression analyses including the Ki67 index into account were undertaken, a significant interaction between AR-IR and ErbB2-IR with respect to their association with the tumour stage was found. This would suggest that the association of the parameters with the tumour stage is rather complex: no association of ErbB2-IR *per se* with the tumour stage; a negative association for AR-IR (i.e. where a lower AR-IR is associated with a higher tumour stage); a positive association for the rate of cell proliferation as measured by the Ki-67 index; and a positive influence whereby a high AR-IR and a high ErbB2 are associated with a higher tumour stage.

In the cases followed by expectancy after diagnosis, an ErbB2-IR above the optimal (Youden index) cut-off was associated with a poorer prognosis than for cases with an ErbB2-IR below this value. The prognostic value of ErbB2-IR in Pca has been the subject of considerable research, and in their meta-analysis, Neta et al. [10] reported a pooled relative risk of 1.86 (Mantel-Haenszel Fixed RR, 95% confidence interval 1.59–2.19) for cases with moderate-high ErbB2-IR compared to those with none-low immunostaining. In our study, the Mantel-Haenszel Hazard ratio was 1.997 (95% confidence interval 1.238–3.220, calculated from the data shown in Fig. 4A) using the optimal Youden cut-off. This is in reasonable agreement with the literature. However, we feel that tumour ErbB2-IR has limited value as a clinical marker to aid treatment decisions, for three reasons: firstly, its magnitude is limited given that the area under the ROC curve, albeit significant, is a meagre 0.60 (values ≤0.75 have been described as “not clinically useful” [32]). Secondly, it does not provide additive information to that given by the Gleason score or by Ki67, a marker with a higher area under the ROC curve. Finally, it has no significant prognostic value for cases with Gleason score 6–7, the cases where treatment decisions are the most difficult. This latter finding is consistent with the large study of Minner et al. [10] who evaluated the prognostic value of ErbB2-IR in tumour samples obtained at radical prostectomy and with biochemical PSA recurrence as event. In their multivariate Cox proportional hazards analysis with Gleason grade, preoperative PSA level and tumour stage as co-variates, ErbB2-IR did not provide significant additive information (the Exp(B) value for positive ErbB2-IR [n>1400] compared to negative ErbB2-IR [n>300] was 0.91, 95% confidence interval 0.81–1.01, P = 0.099).

One important caveat should be made, namely that the prognostic value of ErbB2 may be dependent upon the time upon sampling and upon the subsequent treatment of the patients. In the present study, the samples were taken at diagnosis, and the prognostic determinations were made using the patients who were followed by expectancy, this being the standard treatment protocol at the time. Given the effects of androgen deprivation upon ErbB2 expression (present study and [8], [9], [33]), the prognostic value of ErbB2 measured following standard androgen therapy may be different to that seen here.

A final note concerns the role of ErbB2 in Pca. The present study was designed to determine whether or not tumour ErbB2-IR per se is a clinically useful marker of Pca disease outcome, rather than to shed light upon the involvement of the gene product in the pathogenesis of the disease. ErbB2 forms heterodimers with other members of its family (such as EGFR) and the present results do not rule out the possibility that patients with an over expression of a particular heterodimer subset are more at risk than other patients [34]. Data for the phosphorylated (active) form of EGFR is available for the tumor microarray [35] and tumor ErbB2-IR provides additional prognostic information to that supplied by tumor pEGFR-IR in a bivariate Cox proportional hazards regression analysis (see also Tables S2 and S3 for ROC and binary logistic regression data). However, no significant interaction between the two were parameters were seen when the interaction term ErbB2-IR x pEGFR-IR was included in the Cox proportional hazards regression analysis (data not shown). It would clearly be of interest to investigate the potential importance of ErbB2 heterodimers in more detail in future studies.

In conclusion, the three main findings of the present study are: 1) that the association of ErbB2-IR with the Gleason score seen at diagnosis is most likely due to the association of both parameters with the rate of tumour cell proliferation; 2) that although ErbB2 expression at the mRNA level is sensitive to the concentration of circulating androgens, there is no association between ErbB2-IR and AR-IR in prostate cancer. However, the two parameters interact with respect to their association with the tumour stage; and 3) that ErbB2 is not a clinically useful prognostic marker for predicting disease-specific survival as adjudged by its performance in the tissue microarray used here.

## Supporting Information

Table S1 (DOCX)

Table S2 (DOCX)

Table S3 (DOCX)
