# Supplementary material for: ErbB2 Receptor Immunoreactivity in Prostate Cancer: Relationship to the Androgen Receptor, Disease Severity at Diagnosis and Disease Outcome
Source: PLoS One. 2014 Sep 12;9(9):e105063. doi: 10.1371/journal.pone.0105063 (PMC4162542; doi:10.1371/journal.pone.0105063)
Supplement: Table S1 — (DOCX) [file pone.0105063.s001.docx]

**Table S1.** Logit and odds (P) ratio for the ordinal regression analyses summarised in Table 3.

|  | logit(P[Y≥2]) | logit(P[Y≥3]) | logit(P[Y≥4]) |
| --- | --- | --- | --- |
| *Main effects model* |  |  |  |
| AR median, ErbB2 median | -0.159 | -1.587 | -3.910 |
| AR minimum, ErbB2 median | 1.164 | -0.264 | -2.587 |
|  |  |  |  |
| *Interaction model* |  |  |  |
| AR median, ErbB2 median | -0.169 | -1.610 | -3.927 |
| AR median, ErbB2 minimum | -0.474 | -1.916 | -4.233 |
| AR median, ErbB2 maximum | -0.016 | -1.457 | -3.775 |
| AR minimum, ErbB2 median | 1.112 | -0.330 | -2.647 |
|  |  |  |  |
|  | P[Y≥2]/P[Y<2] | P[Y≥3]/P[Y<3] | P[Y≥4]/P[Y<4] |
| *Main effects model* |  |  |  |
| AR median, ErbB2 median | 0.853 | 0.205 | 0.020 |
| AR minimum, ErbB2 median | 3.201 | 0.768 | 0.075 |
|  |  |  |  |
| *Interaction model* |  |  |  |
| AR median, ErbB2 median | 0.845 | 0.200 | 0.020 |
| AR median, ErbB2 minimum | 0.622 | 0.147 | 0.015 |
| AR median, ErbB2 maximum | 0.984 | 0.233 | 0.023 |
| AR minimum, ErbB2 median | 3.040 | 0.719 | 0.071 |

Y refers to the response level, where Y(1) is the reference level. Minimum, median and maximum values för ErbB2-IR were 1, 3 and 4, respectively. Minimum and median values for AR-IR were 0.2 and 50%, respectively. Ki67-IR was held at the median value (2.63%) throughout.
