# Supplementary material for: ErbB2 Receptor Immunoreactivity in Prostate Cancer: Relationship to the Androgen Receptor, Disease Severity at Diagnosis and Disease Outcome
Source: PLoS One. 2014 Sep 12;9(9):e105063. doi: 10.1371/journal.pone.0105063 (PMC4162542; doi:10.1371/journal.pone.0105063)
Supplement: Table S2 — (DOCX) [file pone.0105063.s002.docx]

**Table S2.** ROC area under the curves for combinations of markers.

| Marker | AUC | 95% CI | | P-value | Event 0 | Event 1 |
| --- | --- | --- | --- | --- | --- | --- |
|  |  | Lower | Upper |  |  |  |
| Ki67 | 0.75 | 0.69 | 0.81 | < 0.0001 | 193 | 68 |
| AR | 0.67 | 0.59 | 0.75 | < 0.0005 | 178 | 61 |
| pAkt | 0.61 | 0.53 | 0.70 | < 0.05 | 135 | 54 |
| ErbB2 | 0.60 | 0.52 | 0.68 | < 0.05 | 167 | 66 |
|  |  |  |  |  |  |  |
| Ki67 + AR | 0.72 | 0.64 | 0.79 | < 0.0001 | 174 | 59 |
| Ki67 + pAkt | 0.69 | 0.61 | 0.77 | < 0.0001 | 133 | 53 |
| Ki67 + ErbB2 | 0.65 | 0.57 | 0.73 | < 0.0005 | 163 | 64 |
| AR + pAkt | 0.71 | 0.63 | 0.80 | < 0.0001 | 125 | 49 |
| AR + ErbB2 | 0.70 | 0.62 | 0.77 | < 0.0001 | 148 | 57 |
| pAkt + ErbB2 | 0.57 | 0.48 | 0.66 | 0.17 | 127 | 52 |
|  |  |  |  |  |  |  |
| Ki67 + AR + pAkt | 0.76 | 0.68 | 0.84 | < 0.0001 | 122 | 48 |
| Ki67 + AR + ErbB2 | 0.72 | 0.64 | 0.80 | < 0.0001 | 145 | 55 |
| Ki67 + pAkt + ErbB2 | 0.63 | 0.54 | 0.72 | < 0.01 | 125 | 51 |
| AR + pAkt + ErbB2 | 0.68 | 0.60 | 0.77 | < 0.0005 | 117 | 47 |
|  |  |  |  |  |  |  |
| Ki67 + AR + pAkt + ErbB2 | 0.70 | 0.62 | 0.79 | < 0.0001 | 115 | 46 |

A fifteen year cut-off was used. Values were normalised to that the highest observed score for the parameter in question was set to 100%. In the case of the androgen receptor (AR), where a low score is associated with a poor prognosis, the values were then subtracted from 100%, i.e. the scores were reversed. Events 0 and 1 refer to patients who were still alive / died from other causes and patients who died of prostate cancer during the follow up period, respectively.
