# Supplementary material for: ErbB2 Receptor Immunoreactivity in Prostate Cancer: Relationship to the Androgen Receptor, Disease Severity at Diagnosis and Disease Outcome
Source: PLoS One. 2014 Sep 12;9(9):e105063. doi: 10.1371/journal.pone.0105063 (PMC4162542; doi:10.1371/journal.pone.0105063)
Supplement: Table S3 — (DOCX) [file pone.0105063.s003.docx]

**Table S3.** Binary logistic regression analysis for the normalised marker data.

|  | no. cases event 0/1 | Nagelkerk pseudo r^2^ | Exp B | 95% CI | | P-value | Hosmer- Lemeshow | % Event 1 predicted |
| --- | --- | --- | --- | --- | --- | --- | --- | --- |
|  |  |  |  | lower | upper |  |  |  |
| *One marker* | |  |  |  |  |  |  |  |
| Ki67 | 193/68 | 0.10 | 1.046 | 1.022 | 1.070 | < 0.0005 | < 0.0001 | 11.8 |
| AR | 178/61 | 0.10 | 1.029 | 1.014 | 1.043 | < 0.0001 | 0.84 | 9.8 |
| pAkt | 135/54 | 0.038 | 1.025 | 1.002 | 1.047 | < 0.05 | 0.10 | 0 |
| ErbB2 | 167/66 | 0.039 | 1.020 | 1.004 | 1.035 | < 0.05 | 0.054 | 0 |
|  |  |  |  |  |  |  |  |  |
| *Two markers* | |  |  |  |  |  |  |  |
| Ki67 | 174/59 | 0.16 | 1.049 | 1.019 | 1.079 | < 0.005 | 0.87 | 16.9 |
| AR |  |  | 1.025 | 1.010 | 1.041 | < 0.005 |  |  |
|  |  |  |  |  |  |  |  |  |
| Ki67 | 133/53 | 0.15 | 1.057 | 1.024 | 1.092 | < 0.001 | 0.71 | 22.6 |
| pAkt |  |  | 1.009 | 0.985 | 1.034 | 0.48 |  |  |
|  |  |  |  |  |  |  |  |  |
| Ki67 | 163/64 | 0.10 | 1.037 | 1.013 | 1.062 | < 0.005 | 0.22 | 15.6 |
| ErbB2 |  |  | 1.013 | 0.996 | 1.029 | 0.13 |  |  |
|  |  |  |  |  |  |  |  |  |
| AR | 125/49 | 0.15 | 1.032 | 1.016 | 1.049 | < 0.0001 | 0.86 | 22.4 |
| pAkt |  |  | 1.020 | 0.995 | 1.045 | 0.11 |  |  |
|  |  |  |  |  |  |  |  |  |
| AR | 148/57 | 0.14 | 1.030 | 1.014 | 1.045 | < 0.0005 | 0.77 | 15.8 |
| ErbB2 |  |  | 1.020 | 1.003 | 1.038 | < 0.05 |  |  |
|  |  |  |  |  |  |  |  |  |
| pAkt | 127/52 | 0.029 | 1.015 | 0.991 | 1.040 | 0.22 | 0.32 | 0 |
| ErbB2 |  |  | 1.008 | 0.989 | 1.028 | 0.40 |  |  |
|  |  |  |  |  |  |  |  |  |
| *Three Markers* | |  |  |  |  |  |  |  |
| Ki67 | 123/48 | 0.19 | 1.045 | 1.008 | 1.083 | < 0.05 | 0.37 | 22.9 |
| AR |  |  | 1.030 | 1.012 | 1.047 | < 0.001 |  |  |
| pAkt |  |  | 1.008 | 0.981 | 1.035 | 0.58 |  |  |
|  |  |  |  |  |  |  |  |  |
| Ki67 | 145/155 | 0.16 | 1.036 | 1.005 | 1.069 | < 0.05 | 0.91 | 21.8 |
| AR |  |  | 1.026 | 1.010 | 1.043 | < 0.005 |  |  |
| ErbB2 |  |  | 1.013 | 0.995 | 1.032 | 0.15 |  |  |
|  |  |  |  |  |  |  |  |  |
| AR | 117/47 | 0.15 | 1.033 | 1.016 | 1.050 | < 0.0005 | 0.48 | 23.4 |
| pAkt |  |  | 1.009 | 0.981 | 1.038 | 0.53 |  |  |
| ErbB2 |  |  | 1.012 | 0.990 | 1.033 | 0.29 |  |  |
|  |  |  |  |  |  |  |  |  |
| *Four Markers* | |  |  |  |  |  |  |  |
| Ki67 | 115/46 | 0.17 | 1.033 | 0.995 | 1.073 | 0.090 | 0.47 | 19.6 |
| AR |  |  | 1.029 | 1.012 | 1.047 | <0.005 |  |  |
| pAkt |  |  | 1.001 | 0.973 | 1.031 | 0.93 |  |  |
| ErbB2 |  |  | 1.006 | 0.984 | 1.029 | 0.61 |  |  |

The data with a 15 year cut-off, was normalised as described in Table S1, and the outcome measures were event 0 or 1. The Nagelkerke pseudo r^2^ is a goodness of fit measure. Exp(B) indicates the predicted odds ratio. Note that the value for AR is above unity because the scale has been reversed. The Hosmer-Lemeshow test is a measure of model adequacy; a significant P value suggesting that the model is not adequate. “% Event 1 predicted” indicates how many (in percentage) of the cases who died as a result of their cancer (event 1) were correctly predicted, the initial value being 0%.
